# Environmental Factors Influence Plant Vascular System and Water Regulation

**DOI:** 10.3390/plants8030065

**Published:** 2019-03-15

**Authors:** Mirwais M. Qaderi, Ashley B. Martel, Sage L. Dixon

**Affiliations:** 1 Department of Biology, Mount Saint Vincent University, 166 Bedford Highway, Halifax, NS B3M 2J6, Canada; sage.dixon3@msvu.ca; 2 Department of Biology, Saint Mary’s University, 923 Robie Street, Halifax, NS B3H 3C3, Canada; ashley.martel@smu.ca

**Keywords:** climate change, drought stress, elevated carbon dioxide, environmental factors, higher temperature, plants, transpiration stream, vascular cambium

## Abstract

Developmental initiation of plant vascular tissue, including xylem and phloem, from the vascular cambium depends on environmental factors, such as temperature and precipitation. Proper formation of vascular tissue is critical for the transpiration stream, along with photosynthesis as a whole. While effects of individual environmental factors on the transpiration stream are well studied, interactive effects of multiple stress factors are underrepresented. As expected, climate change will result in plants experiencing multiple co-occurring environmental stress factors, which require further studies. Also, the effects of the main climate change components (carbon dioxide, temperature, and drought) on vascular cambium are not well understood. This review aims at synthesizing current knowledge regarding the effects of the main climate change components on the initiation and differentiation of vascular cambium, the transpiration stream, and photosynthesis. We predict that combined environmental factors will result in increased diameter and density of xylem vessels or tracheids in the absence of water stress. However, drought may decrease the density of xylem vessels or tracheids. All interactive combinations are expected to increase vascular cell wall thickness, and therefore increase carbon allocation to these tissues. A comprehensive study of the effects of multiple environmental factors on plant vascular tissue and water regulation should help us understand plant responses to climate change.

## 1. Understanding the Effects of Climate Change on Plant Water Status

Atmospheric carbon dioxide (CO_2_), air temperature, and drought are the main components of climate change [1,2]. As reported, global mean surface air temperatures have already risen by 1–2 °C, and are expected to rise another 2–3 °C by 2100 [2], with one of the greatest degrees of warming projected for Northern latitudes [3]. Since the industrial revolution, atmospheric CO_2_ has been steadily increasing, with a current concentration of over 400 µmol mol^−1^, and a projected concentration of 700 µmol mol^−1^ by the end of the century [2,3,4]. While one-third of the world is currently facing some form of water deficit, water availability is predicted to further decrease by 20%–70% [5], leading to increased incidences of drought and a need for improvements in plant water use efficiency (WUE). In order to properly adjust to climate change, photosynthetic processes and WUE of plants must both be increased substantially [6]. Plant photosynthesis depends on both atmospheric CO_2_ and the presence of sunlight, and along with related processes, such as transpiration and respiration, it is sensitive to changes in global climate [7]. Uptake of CO_2_ occurs through stomata, small pores on the epidermal cells of plants [8,9]. Guard cells surround each stoma and regulate uptake of CO_2_ and release of water vapour by opening and closing of the stomata [8,10]. Stomata are sensitive to external factors, and water transport and stomatal regulation both depend strongly on the development of vascular tissues [11], hydraulic conductance, and water potential [12]. Plant water transport can be disrupted by environmental factors [13], which adversely affect plant metabolism, growth, or reproduction [14,15].

Many studies have examined the individual and two-way interactive effects of temperature, CO_2_, and drought on plant functions [5,7,16,17,18], such as transpiration stream and photosynthesis [19,20,21,22,23,24,25]; however, few studies have considered the effects of all these factors together [5,16,17,26,27,28,29,30,31]. Studies on the effects of these factors on the development of vascular tissue are particularly scarce. In a changing climate, it is important to understand how environmental factors interact and affect plants. The in-depth effects of these three factors on vascular tissue and water regulation have yet to be examined [5]. The main objectives of this review are: (i) to synthesize available information regarding the effects of the main climate change components on plant vascular system and (ii) to predict possible changes in vascular tissue and water regulation of plants, as they are influenced by multiple environmental factors. In this review, we first discuss plant transpiration and its regulating factors, and then the individual and interactive effects of three components of climate change on plants and the form and function of their vascular system.

## 2. Plant Transpiration and Its Regulating Factors

Each year, plants contribute 32 × 10^3^ billion tonnes of water vapour to the atmosphere as a result of transpiration, accounting for 30% of annual precipitation [32]. For each molecule of CO_2_ gained through the stomata, a plant loses approximately 200 to 400 molecules of water [9], and therefore water uptake is critical for proper plant functioning. Transpiration creates tension, leading to a tug-of-war process that drives a continuous stream of water molecules from the roots to the leaves, known as the cohesion-tension theory [33,34]. This process pulls water through the transpiration stream, where it mainly evaporates from the stomatal pore [9,35]. Although most gas exchange occurs through stomata when they are open, the cuticle controls the transpiration rate when stomata are closed [36], for instance, due to drought stress or elevated CO_2_ concentration. Water movement is also driven by water potential, with water moving from an area of higher potential to an area of lower potential [37]. Transpiration can occur at high rates and, therefore, water must effectively be replaced through root uptake and transported through vascular tissue; this is a passive process requiring little energy [9].

### 2.1. Plant Vascular Structure and Function

Water transport through xylem is over a million times more efficient than water transport through plasmodesmata of parenchyma [9]. Once water reaches the xylem, it enters conducting elements of either conifer tracheids or angiosperm vessels [38], and flows upwards through the stem to the leaves [35]. The conduit diameter of xylem gets smaller and tapered with plant height, indicating the widening aspect of xylem anatomy from apex to the base of plant [35]. Plants that have an increased number of xylem conduits per cross-sectional area can maintain hydraulic conductance by reducing effects of path length [35]. Xylogenesis, the process by which conduits are formed through programmed cell death, results in a long-distance, low-resistance pathway composed of non-living cells [39] that act as a water pipeline [35,40]. Since it is composed of non-living cells that cannot acclimate to the changing environmental factors, conditions at the timing of xylogenesis are important.

Earlier studies have suggested that vascular anatomy is important in plant acclimation potential. For example, common oak (*Quercus robur* L.) trees that died in response to a widespread drought had a greater xylem vessel diameter than trees that survived, so these anatomical traits may increase drought susceptibility [41]. Also, vegetation shifts due to climate change lead to more ecological drought [42] and can affect plants and their vascular system in the new environment.

### 2.2. Vascular Cambium and Plant Growth

When active, vascular cambium undergoes cell division to contribute to secondary growth of xylem and phloem, representing the largest carbon sink in vascular plants [3,11]. The cambial zone refers to all layers of meristematic cells and their derivatives between the xylem and phloem. Increased cambial division leads to increased production of xylem biomass [11]. Developing cells have different stages of primary and secondary cell wall formation and lignification [43]. During primary growth, procambial cells promote upward growth of vascular tissue [11]. Patterns of radial growth are positively correlated with the width of the cambial zone [44], which varies among individuals of a species [43].

Cambial development can also be controlled by interactions of phytohormones, such as auxins, gibberellins, cytokinins, and ethylene [9,11,44]. For example, a low concentration of ethylene can have a stimulatory effect on cambial cell division in young shoot of white leadtree (*Leucaena leucocephala* (Lam.) de Wit.) [45]. However, a low concentration of cytokinin signaling is the primary basis for the impaired cambial growth of poplar (*Populus trichocarpa* Torr. and A. Gray ex. Hook.) [46]. Since environmental factors can influence hormone levels and transport [47,48], cambial development may be indirectly affected; for example, drought reduces transport of cytokinins from root to shoot but increases transport of an ethylene precursor [48].

### 2.3. Plant Hydraulic Conductance

The water pressure gradient on either side of the stomata generates transpiration, and passively lifts water from the soil into the roots. As water is lost due to transpiration, adequate hydraulic conductance is required to replace the transpired water [9]. Plant hydraulic conductance can be calculated as water flow divided by the difference in pressure or water potential [49]. Considering the relationship between transpiration and hydraulic conductance, anatomical traits that influence stomatal regulation can also affect hydraulic conductance. Leaf anatomical parameters, such as vein characteristics and mesophyll anatomy, can help determine the sites of water evaporation and flow resistance patterns, with a large impact on stomatal regulation [50]. Small variations in leaf water potential can affect stomatal regulation and water flow coordination [50]. Recent work suggests that the transport of water vapour between mesophyll and epidermis may contribute to the regulation of stomatal movement [12,51,52].

Another anatomical feature that influences hydraulic conductance is the lignin content of cell wall. In conducting elements, the secondary cell walls contain significant amounts of lignin, which is required for structural support and affects water transport [9]. For example, in poplar (*Populus* spp.), decreased xylem conductance was associated with reduced lignin content [53]. Meanwhile, suberin, a fatty polymer, can also influence the movement of water by preventing uncontrolled water movement in the roots, where there are layers of differentiated Casparian bands [54]. The forming of these bands impede horizontal water flow via the apoplastic pathway [55]; their deposition is extremely specific [54], and may be controlled by environmental factors [56].

Hydraulic conductance can be disrupted when environmental factors, such as extreme temperature and drought, create an unsustainable level of xylem tension, leading to a process called cavitation—breaking of water column [57]. This leads to separation of air from water, resulting in a gas bubble called an embolism that blocks the conduit and prevents water movement [9,57,58]. Besides the tension-driven embolism, the freeze–thaw events can also lead to embolism; in this case small gas bubbles are formed in the frozen liquid [18]. Moreover, changes in sap chemistry (e.g., due to pathogen infection) can induce embolism [59]. All these stress conditions increase the frequency of embolism, leading to decreased plant productivity [18]. In order to cope with embolism, plants can re-route water through nearby xylem, create new xylem [9,57], or refill vessels to force the air bubbles to dissolve in water [60]. Refilling requires hydraulic isolation from tensions, which prevent embolism repair; as shown hydraulic conductivity in the xylem can be restored in the presence of tensions in the bulk xylem [60]. Aquaporins also play a role in the repair of embolism during its refilling [61]. Although aquaporins can respond to environmental factors, they will not be discussed in detail in this paper since several recent reviews have already focused on them [37,62,63,64,65]. Failure to fix embolism can result in a reduced hydraulic capacity, limited photosynthesis, or runaway embolism and even plant death [9,66]. Failure of hydraulic conductance is one of the main causes of plant mortality under drought conditions [67].

## 3. Environmental Factors and Plant Water Status

Most environmental stress factors have common effects on plants, including inhibition of growth, reduced photosynthesis, hormone fluctuations, and accumulation of stress-related compounds. Often, these changes occur as a result of dehydration caused by an imbalance between water uptake in the roots and water loss through leaf transpiration [68], with local weather extremes holding the highest impact on plant survival and productivity. In particular, xylem physiological function is highly vulnerable as tree survival depends on its ability to sustain water supply to the tree crown under variable environmental conditions [18]. The first line of defense against plant dehydration is often stomatal closure, and since stomatal conductance and water transport are coupled, changes in one will affect the other, resulting in changes in overall photosynthetic processes [18].

Environmental factors have an important effect on the initiation and differentiation of vascular cambium. The rate of cambial cell division and, in turn, xylem development, is correlated with temperature, rainfall, and humidity [44,69]. Higher rainfall leads to cambial reactivation, and subsequent differentiation of xylem vessel elements, whereas these processes are negatively correlated with temperature. Cambial activity of woody plants is very sensitive to water deficits and drought decreases or delays cell division of vascular cambium by reducing turgor pressure of cambial cells, leading to reduced plant growth [44] (see Table 1).

Environmental stress tolerance can be increased by accumulation of metabolites, such as glycinebetaine [76]. It is critical to understand how climate change components—high temperature, elevated CO_2_ and drought—affect growth, vascular systems, and water status of plants.

### 3.1. Thermal-Related Responses of Plant Growth and Vascular System

Higher temperatures decrease photosynthesis and WUE but increase transpiration and stomatal conductance, leading to a shorter growth period and faster development [77]. In C_3_ plants, short-term increases in air temperature can affect photosynthesis by altering either the catalytic properties of Rubisco (ribulose-1,5-bisphosphate carboxylase/oxygenase) or the ratio between CO_2_ and O_2_ at Rubisco active sites, resulting in increased photorespiration. Most plants can undergo thermal acclimation, shifting their photosynthetic temperature optimum and maximizing photosynthesis at higher growth temperatures, but large changes in temperature may damage the photosynthetic apparatus [7] or increase transpiration to an extent where it leads to drier soils in already water-limited habitats [78]. Acclimation of plants results in increased stability of photosystem membrane, expression of heat-stable Rubisco enzymes, production of heat-shock proteins, decreases in respiration [79], and decreased stomatal conductance due to a decline in intercellular CO_2_ [7]. Rising temperatures are expected to increase respiration and photorespiration, but these may be minimized through acclimation or down-regulation of photosynthetic capacity [4]. Earlier studies have shown that higher temperature increases both outside-xylem hydraulic conductance [12] and mesophyll conductance [4], increasing gas-phase conductance [12], which can sustain turgor pressure within guard cells and increase transpiration rates [50].

In seasonal climates, temperature plays an essential role in vascular development of woody plants, as earlier warming of temperature induces an earlier onset of the growth season through stimulation of cambial activity [16,24,80,81]. Increased temperature of at least 6–8 °C at the onset of the growing season stimulates xylogenesis [29], whereas initiation of advanced formation of phloem, compared to xylem cells, requires lower temperature, following which it is endogenously controlled [24,81]. In woody plants, higher temperature can increase (e.g., in Khasi pine) or decrease (e.g., in Momi fir) tracheid diameter, depending on the species [16,20] (see Table 1). Increased diameter of xylem vessel or tracheid increases the efficiency of water transport [9]. While earlier cambial activity, due to early temperature warming, may be positive and results in increased wood biomass production and water transport efficiency, the cambium may also be at greater risk from frost damage if there are any sudden drops in temperature after the initial early spring warming [80]. In herbaceous plants, such as potato, increased temperature can lead to enlarged and deformed vessel cells and improper phloem division [72] (see Table 1). These changes can reduce crop yields as enlarged xylem negatively affects the phloem by putting mechanical pressure on its cells, resulting in decreased sugar translocation [72,82].

Plants under extreme heat stress can be susceptible to vascular damage. Typically, plant cells have a threshold temperature, after which vascular cell death is observed; however, cell death can also occur due to prolonged heat stress at a level below the threshold [83]. Heat stress can increase vulnerability to cavitation by changing sap surface tension and deforming conduit cell walls [84]. Larger plants with thicker epidermis or stem diameter can be protected from damage to their vascular bundles under high temperature conditions, but since plant size, along with stem diameter, has been shown to decrease under increased temperature [1], this may leave plants more susceptible to prolonged heat stress.

### 3.2. CO_2_-Dependent Responses of Plant Growth and Vascular System

In contrast to thermal effects, it is widely accepted that elevated CO_2_ increases plant growth, and may help mitigate the negative effects of other environmental stressors [9,28,31,85,86,87]. Elevated CO_2_ decreases stomatal conductance, alters capacity for carboxylation, and results in accumulation of photoassimilates [86,87]; it also increases water potential and leads to a reduced transpiration stream, higher WUE, and increased resistance to cavitation [12,17,85].

Plants grown under elevated CO_2_ have a higher transpirational demand and reduction of investment into plant cell walls [17]. Overall, net CO_2_ assimilation is expected to increase to a certain extent due to increased atmospheric CO_2_ concentration. This increase occurs as a result of both increased CO_2_ available for the Rubisco active site and decreased transpiration [88]. In response to increasing atmospheric CO_2_, stomatal conductance, which increases WUE, is often reduced [4]. This can increase leaf temperature, leading to increased water transport through the transpiration stream. In many plants, elevated CO_2_ has been shown to increase belowground biomass, allowing for greater root area for water uptake [86]. Growth under elevated atmospheric CO_2_ leads to a reduction in stomatal density in plants with passive stomatal control [10,17]. In short-term experiments, elevated CO_2_ generally increases photosynthesis and decreases transpiration, but under long-term exposure photosynthetic processes acclimate. It is commonly found that elevated CO_2_ can offset the negative effects of decreased Rubisco activity; as such, photosynthetic acclimation typically leads to a decreased photosynthetic capacity as opposed to activity [88,89]. Therefore, plants grown at elevated CO_2_ have a decreased ability to meet water demand through xylem water transport [17]. A decrease in stomatal aperture and, in turn, stomatal conductance was observed in royal fern (*Osmunda regalis* L.) in response to unnaturally high CO_2_ concentration of 1500 µmol mol^−1^ [10], but FACE (free-air CO_2_ enrichment) experiments using soybean (*Glycine max* (L.) Merr.) did not show this response, and there may be different levels of acclimation in different species [4].

Some evidence suggests that woody plants may have reached a saturation level of CO_2_ [90], whereas other studies have predicted a positive effect of CO_2_ on plant growth and development [4,10,17,87]. In some species, small increases in net CO_2_ assimilation may be offset by similar increases in respiration, and increases in CO_2_ may not result in any change in carbohydrate formation [18].

Effects of increasing CO_2_ on vascular characteristics remain largely unstudied; however, CO_2_ may induce a plastic response that could allow plants to synchronize water transport with hydraulic demand [91]. Increased hydraulic demand may result in larger conduits, higher conduit density, or greater xylem size in reference to stem cross-sectional area. When grown at elevated CO_2_, plants have been shown to have larger xylem conduits than those grown at ambient CO_2_, with a reduced ratio of conduit wall thickness to diameter [17]. This may be beneficial because increased conduit diameter results in a four-fold increase in leaf transpiration and water supply. On the other hand, larger leaf area with thinner leaves and larger stomata [17] could increase vulnerability to cavitation, as insufficient carbon supply may lead to increased conduit membrane porosity and likelihood of air entry [91].

Elevated CO_2_ is likely to result in greater fortification of xylem conduits via an increase in double wall thickness [92], and, in order to reduce sensitivity to other environmental stress factors, plants can develop more robust pit membranes to reduce vulnerability to embolism [91]. In woody plants (e.g., Norway spruce), elevated CO_2_ decreases the concentrations of soluble sugar, acid-soluble lignin, and nitrogen [70]. In herbaceous plants (e.g., common bean), elevated CO_2_ decreases vessel density, but increases vessel diameter and embolism [17] (see Table 1). While earlier work has suggested that elevated CO_2_ may mitigate the effects of other environmental stress factors, it remains under debate whether CO_2_ saturation will occur [90]. For instance, sufficient nutrients, such as nitrogen, are likely necessary for long-term increases in growth under conditions of elevated CO_2_ [93]. Regardless of its implications for forestry and agriculture, the effects of elevated CO_2_ on plant vascular tissue remain largely unstudied.

### 3.3. Drought-Related Responses of Plant Growth and Vascular System

Drought stress can lead to photosynthetic inhibition through leaf senescence, reduced growth, and feedback inhibition of photosynthetic enzymes [89] along with initial processes, such as stomatal closure, which occurs as one the earliest responses to drought [94]. Under drought stress, the water cost associated with carbon fixation causes a negative tradeoff as plants may either dehydrate or have a reduced rate of carbon fixation, leading to carbon starvation [18]. A plant is considered to be under drought stress when the soil water content is not replenished by rainfall or irrigation [68]. At the onset of drought stress, stem hydraulic capacitance, transpiration and root water uptake begin to decline [68,95]. Root hydraulic conductance declines, and as soil water content diminishes, roots lack sufficient water supply. Initially, root hydraulic conductance decreases, as the Casparian band potentially reduce the backflow of water from root to soil [68]. Stomatal closure and subsequent reduction in photosynthesis occur rapidly due to reduced carbon fixation and supply of carbon to chloroplasts and Rubisco active sites [1,7,89]. Understanding how plant species respond to drought stress will allow us to better predict the effects of future climate. In North America, warmer temperatures reduce snowpack and subsequently decrease the stream flows of spring and summer, which will increase the length of growing season with incidence of drought stress [85]. A recent climate model has predicted drought-induced reductions in plant hydraulic conductance, canopy transpiration, carbon assimilation, and productivity [96].

Prolonged drought results in decreased photosynthesis, which will result in decreased structural carbohydrates and may promote runaway cavitation [18]. In woody plants, in general, drought stress decreases diameter of vessel and tracheid, thickness of vascular cambium, and delays formation of xylem and phloem, or division of cambium cell [22,24,43,71] (see Table 1). Under drought stress, xylem cavitation is induced, resulting in the formation of embolism and disruption of the transpiration stream—one of the key features affecting plant survival and productivity under drought stress [22]. A comparison between temperate deciduous oaks and Mediterranean evergreen oaks revealed that the latter are less vulnerable to embolism; however, Mediterranean trees that exhibited the most drought stress, had morphological changes, such as narrower vessels, greater pit area, and numerous leaky pits in the inter-vessel [22]. Under prolonged drought, there is an increase in osmotic potential (change in chemical potential of water by solutes) of xylem sap. Similar changes in both leaf and root extracts have been observed in some species, such as grapevine (*Vitis vinifera* L.). This could help contribute to the maintenance of proper water flow, which can be calculated based on the volume flow rate and the force of water flow, including water movement from soil into the roots, under water deficit [97]. When a plant is under drought stress, xylem pressure may change, resulting in stomatal closure through root-to-shoot signaling [37]. Severe drought can lead to complete loss of hydraulic conductance and, in turn, to the desiccation of aboveground tissues and plant mortality [98].

Drought stress may reduce vessel diameter while maintaining vessel density, hydraulic conductance, and conductive area [22]. Precipitation is essential for the formation of latewood in many ring-porous trees [29]. A study showed that during dry periods of the year in Ivory Coast there were no developing cambium cells in teak tree (*Tectona grandis* (L.f.) Kuntze), and at the onset of the rainy season, cambial cells began to swell. Division of phloem cells was observed before division of xylem cells, but xylem cells expanded and differentiated first [43]. In contrast, a study on chestnut (*Castanea* sp.) indicated that production of new xylem and phloem still happened, regardless of an extremely hot and dry month [29], suggesting that there may be differences among species.

Variation in climatic responses could result in a shift in cambial activity. Drought can suppress cambial cell division and inhibits turgor-driven cell enlargement [24]. In dry conditions, plants increase suberization of root apoplastic barriers; for example, root suberization, which is important for water retention, increased in the endodermis but decreased in the sclerenchyma cells of rice [21]. Increased suberization is observed under a number of stress conditions [23]. In the drought-sensitive cultivars of grapevine, more rapid root suberization occurs even under control conditions, as opposed to root suberization in drought-insensitive cultivars of grapevine. Also, under drought stress, suberization occurs closer to the root tip in both cultivars, indicating a faster maturation of root tissue [97]. Hydrotropism can result in deeper roots, with access to deep-water [99]. When deep-water is available, plants have longer root systems with increased root density at such depths, increasing capacity for water transport from soil to shoot as a result of higher frequency of root hairs and increased vessel diameter [100].

Drought can affect other vascular characteristics of trees, such as pit membrane structure, conduit size, and wood density [18]. Drier climates result in smaller pits with thicker and less porous pit membranes, which could be an adaptive response [18]. Xylem anatomical traits have been shown to adjust to drought conditions in trees, as drier conditions result in larger tracheid lumens, thicker cell walls, and a greater number of ray tracheids, increasing efficiency of water transport [18,101]. In angiosperms, dry weather promotes narrow vessel elements, which may reduce the occurrence of embolism; however, this could be a trade-off through decreased xylem transport capacity [18]. While drought stress has been observed to decrease xylem vessel diameter, this may prove adaptive as water transport increases with xylem radius; early-season growth would use less water, conserving it for processes, such as grain filling [23]. In other areas, where conservation is not an issue, increased xylem diameter is considered a desirable characteristic, and is targeted in selective breeding processes [23].

In herbaceous plants, similar to woody plants, drought stress decreases vessel diameter [73,74,75]. Also, cell wall thickening is induced by an increase in lignin polymerization in response to drought that has been observed in common zinnia [73], sugarcane [74], and white clover [75] (see Table 1). Since most current research focuses on woody plants, there is a need for more studies with focus on herbaceous plants [102,103,104].

## 4. Plant Responses to Multiple Environmental Factors

**Figure 1:**
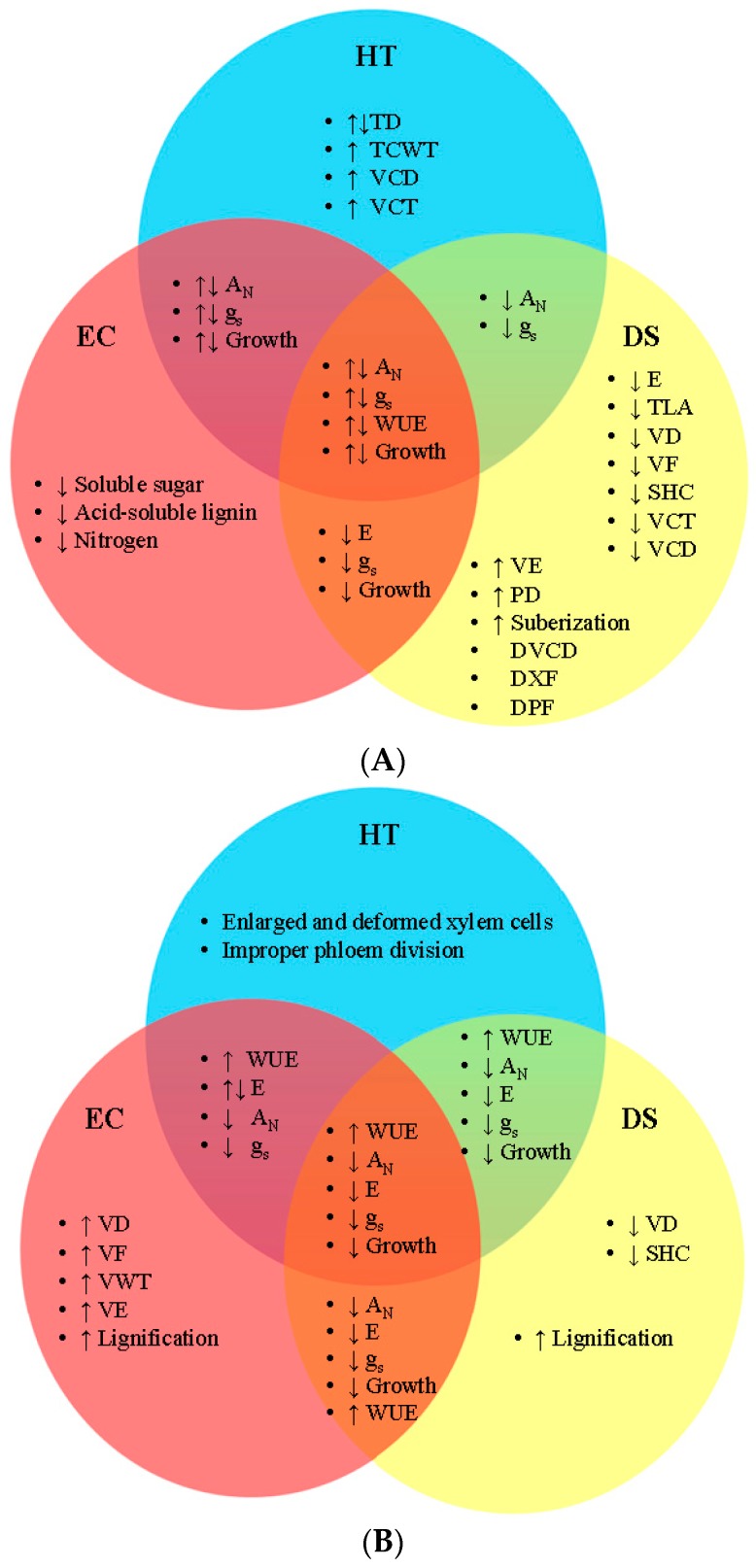
Individual and interactive effects of higher temperature, elevated CO_2_, and drought stress on growth and physiological characteristics of vascular plants. (**A**) Woody plants and (**B**) herbaceous plants. A_N_, net CO_2_ assimilation; DPF, delayed phloem formation; DS, drought stress; DVCD, delayed vascular cambium division; DXF, delayed xylem formation; E, transpiration; EC, elevated CO_2_; g_s_, stomatal conductance; HT, higher temperature; PD, phloem diameter; SHC, shoot hydraulic conductance; TD, tracheid diameter; TCWT, tracheid cell wall thickness; TLA, total leaf area; VCD, vascular cambium division; VCD, vascular cambium division; VCT, vascular cambium thickness; VD, vessel diameter; VE, vessel embolism; VF, vessel frequency (density); VWT, vessel wall thickness; WUE, water use efficiency; ↑, increase; ↓, decrease. Plant properties are based on data from the literature cited in Table 1 and Table 2, and references [85,91] (woody plants) and [115] (herbaceous plants).

In natural habitats, plants experience multiple factors. In this part, earlier findings that have considered the effects of environmental factors on plant growth and physiological activities, and vascular system are summarized, and speculations are made where possible (see Table 2 and Figure 1).

### 4.1. Plant Responses to Temperature and Carbon Dioxide

A recent mathematical model depicts that increased temperature alone is expected to only marginally increase photosynthesis; however, when higher temperature is combined with elevated CO_2_, larger net photosynthesis is expected by up to 50%. On the basis of this model, species that do not acclimate to elevated CO_2_ will experience a greater enhancement of photosynthesis [4], and a greater volume of water will be both lost to the atmosphere and required for root uptake, which may be problematic in water-limited environments.

Plants have been shown to exhibit plastic responses to a number of environmental factors, but many mathematical models fail to take these into account, likely because they remain poorly understood. Photosynthesis and respiration can acclimate in response to temperature and CO_2_, depending on the length and intensity of the stimuli [116]. Reduced transpiration can occur at very high temperatures because Rubisco is temperature-sensitive, slowing down carbon fixation and reducing need for water transport [116].

In woody plants, higher temperature at elevated CO_2_ either increased or decreased net CO_2_ assimilation, stomatal conductance, and growth, as shown in Norway spruce, Scots pine [105], and Tamanqueiro [106]. In herbaceous plants, these two factors together decreased net CO_2_ assimilation and stomatal conductance, increased or decreased transpiration, but increased WUE; the effects varied with species [17,28] (see Table 2 and Figure 1).

Independently, temperature and CO_2_ greatly affected photosynthesis in night-flowering catchfly (*Silene noctiflora* L.). Higher temperature increased transpiration, but did not increase net CO_2_ assimilation or WUE, whereas elevated CO_2_ increased net CO_2_ assimilation and WUE, but decreased transpiration. When these factors were combined, the highest transpiration rate occurred in plants grown under higher temperatures at elevated CO_2_, and these plants were of comparable size to those of control [28]. A study on alfalfa (*Medicago sativa* L.) showed that elevated CO_2_ in combination with increased temperature decreases crude protein but increases plant growth; however, elevated CO_2_ under ambient temperature or under partial irrigation has no effect on plant growth [5]. Studies have yet to examine the effects of these two factors on vascular cambium; however, since plants were of similar size when under higher temperature and elevated CO_2_, this may indicate that vascular cambium may be unaffected, but more in-depth morphological studies are required.

### 4.2. Plant Responses to Temperature and Drought Stress

In combination, higher temperature and increased drought stress lead to reduced crop yield [77,79]. It is projected that negative climate trends, including higher temperature and drought, which associated with rising atmospheric CO_2_, may eventually outweigh the benefits of CO_2_ to plant yield [77] (see Figure 1). When higher temperature is combined with drought stress, the carbon balance of leaves may be offset, leading to an imbalance of photosynthesis and respiration. Fixed carbon is lost in respiration, and overall net photosynthesis and respiration may decline. Full photosynthetic capacity can potentially be restored following restoration of water supply in the absence of irreversible damage [7].

Higher temperature and precipitation may act synergistically on the reactivation of cambial cells and the subsequent formation and differentiation of xylem and phloem cells [16,29]. This would indicate that development of vascular cambium cannot occur without both of the required stimuli—water availability and higher temperature. In combination with increased temperature, reduced water availability may change the timing of cambial cell initiation. Since temperature has the potential to exacerbate the effects of water deficit, earlier suberization may result [97], and vascular tissue may become narrower, resulting in a reduced capacity for xylem-driven water transport [18].

In woody plants (e.g., black poplar), extreme drought stress led to metabolic impairment of photosynthesis. A combination of higher temperature and drought stress decreased net CO_2_ assimilation and stomatal conductance. Drought stress had a greater effect on plant metabolic activities at lower temperature (25 °C) than at higher temperature (35 °C), because maximum photosynthesis was about four fold lower and the maximum rate of Rubisco carboxylation and the apparent maximum rate of electron transport at saturating irradiance were two fold lower at lower temperature than at higher temperature. In addition, plants under lower temperature recovered more slowly than those under higher temperature [7]. In herbaceous plants (e.g., spring wheat), higher temperature with drought stress decreased net CO_2_ assimilation, stomatal conductance, transpiration, and growth, but increased WUE [111] (see Table 2 and Figure 1). In canola (*Brassica napus* L.), plants grown under higher temperatures and drought stress had a lower biomass than plants grown under control conditions—lower temperatures and watering to field capacity. The canola seedlings also had a reduced stem mass [30], which could indicate a reduction of vascular tissues, as they are responsible for the majority of stem girth [11]. Increases in temperature may exacerbate the magnitude of water-stress effect, but this depends, in part, on species and geographical location [5].

### 4.3. Plant Responses to Carbon Dioxide and Drought

It has been suggested that increased atmospheric CO_2_ may lead to improved drought tolerance in plants [117]. However, other studies have suggested that drought may limit the positive effects of elevated CO_2_, regardless of increased WUE [118]. In C_3_ plants, elevated atmospheric CO_2_ could enhance carbon gain while decreasing stomatal conductance [17]. Elevated CO_2_ may directly increase photosynthetic activities, which would contribute to increased growth without water status improvements [85]. Increased photosynthetic rates at elevated CO_2_ have also been shown to be most prominent under drought, and this indicates that elevated CO_2_ could mitigate the negative effects of drought stress. Many of these measurements, however, have been conducted at the leaf level instead of the whole-plant level, which yields less clear-cut results [17]. In a study, elevated CO_2_ increased biomass by 15% in the water-stressed plants [85]. Elevated CO_2_ also increased WUE, but did not increase biomass any more under drought conditions than it did under normal conditions in several riparian tree species [85]; this may be indicative of species variation in response to elevated CO_2_. In many plants, elevated CO_2_ decreases stomatal conductance but increases WUE, and these effects are increased under drier conditions, which may lead to a reduction in plant water demand. In semi-arid environments, transpiration is reduced under elevated CO_2_; this can increase plant growth under dry periods and counteract the negative effects of warming on the supply of available water to plant roots [85]. Under both drought stress and elevated CO_2_, there can be a partial closure of stomata due to increased sub-stomatal CO_2_ concentration [86].

In woody plants (e.g., lemon tree), drought stress combined with elevated CO_2_ decreased both stomatal conductance and transpiration, but had no significant effects on plant biomass [107]. Also, drought stress reduces suberin by up to 70% [19], and the effects of CO_2_ on suberin content has yet to be studied. If CO_2_ does not increase suberin biosynthesis, plants may suffer significant water loss. Since beneficial effects of CO_2_ have been shown to vanish under severe drought stress, CO_2_ fertilization may be unable to compensate for the negative effects of drought stress [18]. In herbaceous plants (e.g., soybean), elevated CO_2_ with drought stress decreased net CO_2_ assimilation, stomatal conductance, transpiration and biomass, but increased WUE [112] (see Table 2 and Figure 1). In a study, the common bean (*Phaseolus vulgaris* L.) plants that were grown under pre-industrial CO_2_ had a higher transpiration rate under moderate drought, and maintained normal net photosynthesis more than plants that were grown under ambient or elevated CO_2_, which had increased WUE and no change in water potential [17]. At elevated CO_2_, the bean plants had a decreased capacity of xylem water transport to meet water demand, and there were stronger drought-induced transpiration limitations [17]. Elevated CO_2_ also increased xylem vessel diameter in bean plants, but drought and the interaction between CO_2_ and drought had no significant effect. Moreover, elevated CO_2_ increases the chance of embolism due to increased vessel diameter [17]. It has been shown that drought-tolerant rice plants, grown at elevated CO_2_, were able to maintain their stomatal conductance under drought conditions by having reduced stomatal density [119]. It remains unknown how CO_2_ affects aspects of vascular cambium initiation and tissue differentiation.

### 4.4. Interactive Effects of Temperature, Carbon Dioxide, and Drought on the Form and Function of Plant Vascular System

Interaction among the three main components of climate change—increased temperature, elevated CO_2_ and drought—can affect plant growth by affecting photosynthesis and dry mass partitioning [120]. Temperature, in combination with elevated CO_2_, has been shown to increase plant growth both under irrigation and drought conditions. In contrast, elevated CO_2_ under ambient temperatures, had no effect on plants in any of the watering regimes, suggesting the importance of interaction among these three factors [5]. Temperature and drought stress cause stomatal closure, and since this reduces carbon fixation, carbohydrate stores could become depleted, leading to tissue starvation. Temperature and CO_2_ both affect tree anatomy and hydraulic conductance, and these changes may result in lower drought tolerance. Beneficial effects of CO_2_ have been shown to vanish under severe drought stress, but these interactions require further study [18].

In woody plants, the interactive effects of these three environmental factors on plant growth and physiology vary with species. For example, interaction among higher temperature, elevated CO_2_, and drought stress increased growth of loblolly pine [108], red ironbark, and Sydney blue gum [110], but decreased that of Monterey pine and Oyster Bay pine [109]. Incidence of embolism might also increase in response to climate change components. Since recovery might depend on the overall plant health, runaway embolism may become more frequent, leading to a higher rate of plant mortality [18]. In herbaceous plants, in general, these three factors decreased net CO_2_ assimilation, stomatal conductance, transpiration, and biomass, but increased WUE, as shown in alfalfa [26,27,113], bird’s-foot trefoil, black medick [114], and canola [1] (see Table 2 and Figure 1). In alfalfa, a decrease in stomatal conductance would indicate a decrease in transpiration. Increased temperature and CO_2_ were shown to decrease Rubisco activity; higher temperature, elevated CO_2_, and drought stress caused Rubisco inhibition [26]. In a study with black knapweed (*Centaurea nigra* L.), plants were grown under varying temperatures, CO_2_, and water regimes. Interaction among these factors significantly affected shoot biomass; plants that were grown under higher temperatures at ambient CO_2_ with drought stress had the lowest shoot mass, whereas plants that were grown under lower temperatures at elevated CO_2_ and watering at field capacity had the highest shoot mass. This indicates that shoot biomass was smaller under a combination of these factors than under the control conditions [31]. A decrease in shoot mass could be indicative of decreased vascular tissue, or larger, less frequent xylem conduits.

**Figure 2:**
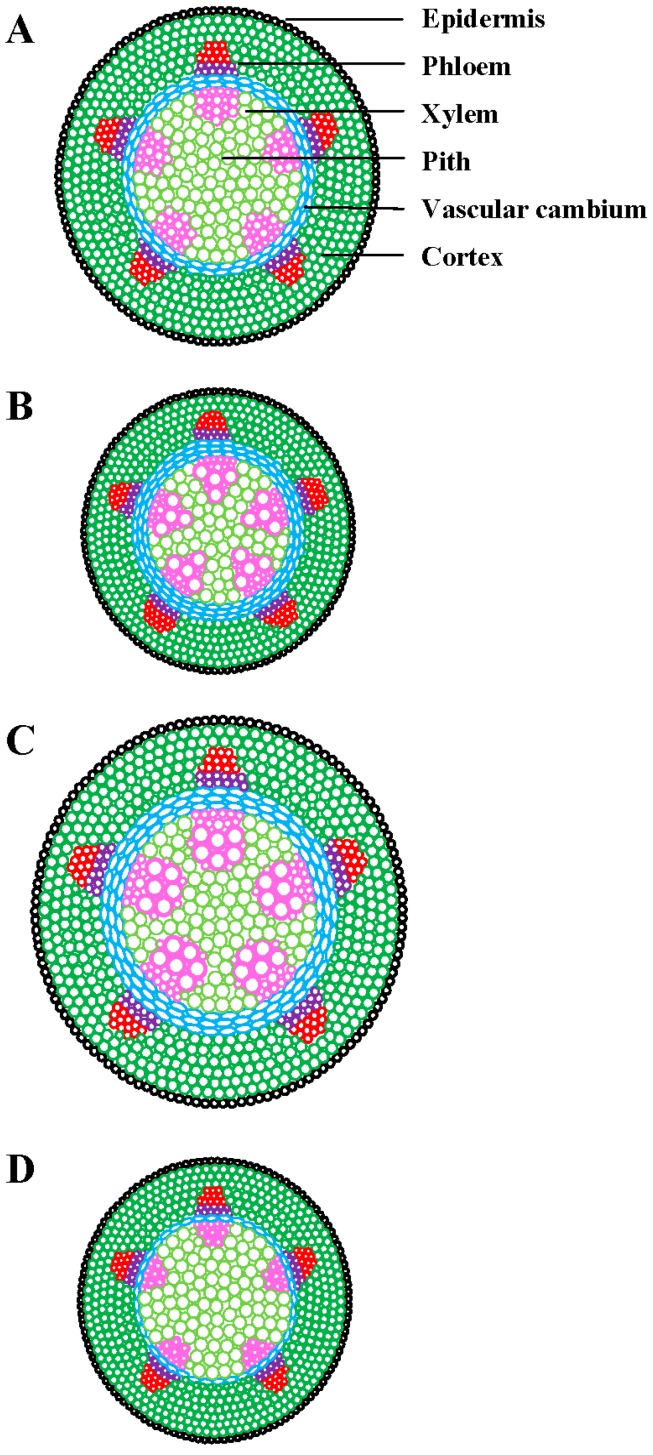
Predicted effects of higher temperature, elevated CO_2_, and drought stress on plant vascular system. (**A**) Control (lower temperature, ambient CO_2_, no drought), (**B**) higher temperature, (**C**) elevated CO_2_, and (**D**) drought stress. For higher temperature, increased xylem diameter and cell wall thickness, increased cambial division and thickness, and decreased overall stem diameter are expected. For elevated CO_2_, increased xylem diameter and density, increased cell wall and cambial thickness, and increased stem diameter are anticipated. For drought stress, decreased xylem diameter and density, increased cell wall thickness, decreased cambial division, and decreased stem diameter are expected. This illustration is based on data from the literature cited in Table 1 and Figure 1.

**Figure 3:**
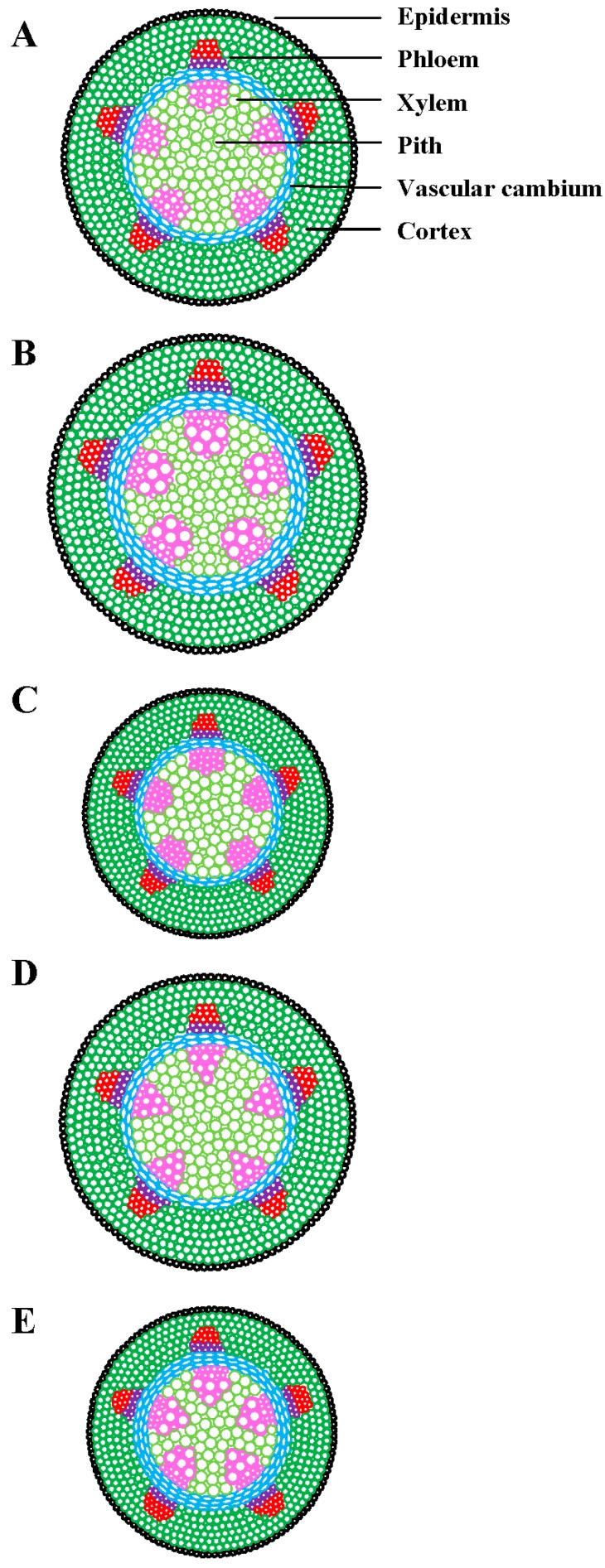
Predicted interactive effects of higher temperature, elevated CO_2_, and drought stress on plant vascular system. (**A**) Control (lower temperature, ambient CO_2_, no drought), (**B**) higher temperature with elevated CO_2_, (**C**) higher temperature with drought stress, (**D**) elevated CO_2_ with drought stress, and (**E**) higher temperature with elevated CO_2_ and drought stress. For higher temperature with elevated CO_2_, increased xylem diameter and density, increased cell wall thickness and cambial thickness, and increased stem diameter are expected. For higher temperature with drought stress, decreased xylem diameter and density, maintained cambial thickness, increased cell wall thickness, and decreased stem diameter are anticipated. For elevated CO_2_ with drought stress, decreased xylem diameter and density, maintained cambial thickness, increased cell wall thickness, and stable stem diameter are expected, unless DS becomes extreme, in which case it may decrease. In the instance of higher temperature with elevated CO_2_ and drought stress, increased vessel wall thickness, increased cambial thickness, and decreased stem diameter are expected. This illustration is based on data from the literature cited in Table 1 and Figure 1.

Climate models have predicted how plants will perform under increased temperature and CO_2_ [5], but not how these factors interact with drought. Acclimation models should examine the potential of leaves that develop under these stress conditions, as they may have a higher acclimation potential than pre-developed leaves that are forced to acclimate under sudden stress induction [116]. On the basis of earlier findings, we predict that the components of global climate change may affect plant vascular system in the future (see Figure 2 and Figure 3).

## 5. Conclusions and Future Directions

In order to predict how plant hydraulics will respond to a changing climate, both structural and functional components, such as plant metabolism, xylem properties, vascular architecture, and leaf size are needed for incorporation into a functional model [35]. Responses to climatic conditions are species-dependent and, therefore, there may be a genetic component to climatic responses, with plant species having differential physiological responses to similar stimuli. Also, studies have shown that the formation of xylem is more sensitive to environmental factors than the formation of phloem [29]. This emphasizes the need for research into the development of vascular tissues, including xylem, phloem, parenchyma and fibers, from the base of plant to shoot apex.

In conclusion, a current challenge in plant physiology is to associate particular structural characteristics of the vascular bundle to specific functions regarding efficiency of water transport [35]. Studies that examine vascular tissue differentiation are rare, and the majority of existing studies look at woody species. More work is needed in determining the effects of environmental factors on vascular development, especially in herbaceous plants. Our lack of understanding makes it difficult to predict how climate change will affect vascular development and the transpiration stream in plants; however, decreased shoot biomass under stress conditions may be indicative of a reduction in vascular tissue. It is essential to understand how plant forms (e.g., vascular tissues) and functions (e.g., photosynthesis and transpiration) will respond to climate change. Optimization of hydraulic efficiency is the first step in ensuring that plants may be better equipped to cope with future climate change.

## Figures and Tables

**Table 1:** Effects of higher temperature, elevated CO_2_, and drought stress on vascular system, relative to control (lower temperature, ambient CO_2_, no drought), in some woody and herbaceous plants. HT, higher temperature; EC, elevated CO_2_; DS, drought stress; ↑, increased; ↓, decreased; -, no effect; NM, not measured.

Environmental Factor	Common Name	SCIENTIFIC NAME	Xylem	Phloem	Vascular Cambium	Structural Polymers	Plant Organ	Experimental Condition	Reference
**Woody plants**									
HT	Khasi pine	*Pinus kesiya* Royle ex. Gordon	↑ tracheid diameter	NM	↑ thickness	NM	Stem	Field	[16]
	Momi fir	*Abies firma* Siebold & Zucc.	↓ tracheid diameter ↑ tracheid cell wall thickness	NM	Induced division	NM	Stem	Field nursery	[20]
EC	Norway spruce	*Picea abies* (L.) Karst.	NM	NM	NM	↓ concentrations of soluble sugar, acid-soluble lignin and nitrogen	Stem	Whole-tree chamber	[70]
DS	Common grape	*Vitis vinifera* L.	↓ vessel diameter ↑ embolism	NM	NM	NM	Stem	Greenhouse	[71]
	Cork oak	*Quercus suber* L.	↓ vessel diameter - vessel density	NM	NM	↑ suberization	Stem	Field	[22]
	European larch	*Larix decidua* Mill.	Delayed formation ↓ tracheid diameter	Delayed formation ↑ diameter	↓ thickness ↓ division	NM	Stem	Field	[24]
	Teak	*Tectona grandis* (L.f.) Kuntze	Delayed formation ↓ vessel thickness	Delayed formation	Delayed division ↓ thickness	- lignification	Stem	Field	[43]
**Herbaceous plants**									
HT	Potato	*Solanum tuberosum* L.	Enlarged and deformed cells	Improper division	NM	NM	Stem	Field	[72]
EC	Common bean	*Phaseolus vulgaris* L.	↑ vessel diameter ↓ vessel density ↑ embolism	NM	NM	NM	Stem	Growth chamber	[17]
DS	Common zinnia	*Zinnia elegans* Sessé & Moc.	↓ vessel diameter	NM	NM	NM	Stem	Greenhouse	[73]
	Sugarcane	*Saccharum* spp.	NM	NM	NM	↑ lignification	Stem	Greenhouse	[74]
	White clover	*Trifolium repens* L.	NM	NM	NM	↑ lignification	Leaf and root	Greenhouse	[75]

**Table 2:** Combined effects of higher temperature, elevated CO_2_, and drought stress on plant growth and gas exchange, relative to control (lower temperature, ambient CO_2_, no drought), in some woody and herbaceous plants. HT, higher temperature; EC, elevated CO_2_; DS, drought stress; A_N_, net CO_2_ assimilation; g_s_, stomatal conductance; E, transpiration; FM, fresh mass; WUE, water use efficiency; ↑, increased; ↓, decreased; -, no effect; NM, not measured; *, *p* < 0.05; **, *p* < 0.01.

Environmental factor	Common Name	Scientific name	Growth/Biomass	A_N_	g_s_	E	WUE	Reference
**Woody plants**								
HT × EC	Norway spruce	*Picea abies* (L.) H. Karst	↓	↓	↓	NM	NM	[105]
	Scots pine	*Pinus sylvestris* L.	↑	↑	↑	NM	NM	[105]
	Tamanqueiro, local name	*Alchornea glandulosa* Poepp. & Endle.	NM	↑	↓	NM	NM	[106]
HT × DS	Black poplar	*Populus nigra* L.	NM	↓ *	↓ *	NM	NM	[7]
EC × DS	Lemon tree	*Citrus limon* (L.) Burm. F. var. ‘Villafranca’	-	-	↓ *	↓ *	NM	[107]
HT × EC × DS	Loblolly pine	*Pinus taeda* L.	↑ * (warm site)	↑ * (June)	↓	NM	NM	[108]
	Monterey pine	*Pinus radiata* D. Don	↓ *	-	-	-	↑ *	[109]
	Oyster Bay pine	*Callitris rhomboidea* R. Br. Ex Rich. & A. Rich.	↓ *	-	-	-	↑ *	[109]
	Red ironbark	*Eucalyptus sideroxylon* A. Cunn ex. Woolls	↑	↓	↓	NM	NM	[110]
	Sydney blue gum	*Eucalyptus saligna* Sm.	↑	↓	↓	NM	NM	[110]
**Herbaceous plants**								
HT × EC	Common bean	*Phaseolus vulgaris* L.	-	↓ *	↓ *	↓ *	↑ *	[17]
	Night-flowering catchfly	*Silene noctiflora* L.	-	-	NM	↑ *	↑	[28]
HT × DS	Spring wheat	*Triticum aestivum* L.	↓ *	↓	↓	↓	↑ *	[111]
EC × DS	Soybean	*Glycine max* (L.) Merr.	↓	↓	↓	↓	↑ **	[112]
HT × EC × DS	Alfalfa	*Medicago sativa* L.	↓ *	↓ *	↓ *	NM	↑ *	[26,27,113]
	Bird’s-foot trefoil	*Lotus corniculatus* L.	↓ * FM	↓ *	↓ *	NM	NM	[114]
	Black medick	*Medicago lupulina* L.	↓ * FM	↓ *	↓ *	NM	NM	[114]
	Canola	*Brassica napus* L.	-	-	↓ *	↓ *	↑ *	[1]
